# Daily torpor in the Djungarian hamster (*Phodopus sungorus*) is orchestrated by the suprachiasmatic nucleus: evidence from immediate early gene mapping and nucleus-specific sequencing

**DOI:** 10.1007/s00360-026-01675-y

**Published:** 2026-05-22

**Authors:** Elena Haugg, Victoria Diedrich, Ceyda Cubuk-Charalampous, Janus Borner, Perry Barrett, Annika Herwig

**Affiliations:** 1 Institute of Comparative Endocrinology and Physiology, Ulm University, Ulm, Germany; 2 Zoological Institute, Hamburg University, Hamburg, Germany; 3 Sackler Institute for Comparative Genomics, American Museum of Natural History, New York, NY USA; 4 The Rowett Institute, University of Aberdeen, Aberdeen, UK

**Keywords:** c-Fos, Gene expression, Metabolism, Siberian hamster, Phodopus sungorus, PVN

## Abstract

Spontaneous daily torpor in the Djungarian hamster is a precisely timed energy-saving strategy that occurs in winter to withstand challenging conditions. It features a transient but pronounced decrease in metabolic rate, body temperature and other physiological parameters. The hypothalamus is thought to remain active to coordinate torpor, but there is limited data with sufficient anatomical resolution to identify nucleus-specific regulatory mechanisms. This study sought to pinpoint hypothalamic regions involved in torpor control and characterize their transcriptional activity using nucleus-specific RNA sequencing. Hamsters were acclimated to short photoperiod and sampled at defined torpor phases (entry, nadir, arousal, post-torpor) and matched times on a torpor-free day. To identify transcriptionally active regions, radioactive in situ hybridization of *c-Fos* was performed. Among all examined nuclei, only the suprachiasmatic nucleus (SCN), the master circadian clock, showed increased *c-Fos* expression during torpor nadir and arousal compared with time-matched normotherms. To assess functional involvement, SCN and paraventricular nucleus (PVN) as key SCN output target, were isolated via laser-capture microdissection from additional animals during torpor entry and arousal for mRNA sequencing. The SCN displayed robust, torpor-specific transcriptional regulation, underscoring its central role in timing daily torpor. The PVN showed fewer but potentially meaningful alterations, including altered expression of aromatase. Enhanced mitochondrial and clock gene activity in the SCN suggests preserved function during hypometabolism, while phase-dependent transcriptional suppression in both nuclei reflects distinct entry–arousal dynamics. Together, these results identify the SCN as a primary coordinator of torpor, with SCN–PVN signalling highlighted as a key pathway for future investigation.

**Supplementary Information:**

The online version contains supplementary material available at 10.1007/s00360-026-01675-y.

## Introduction

Djungarian hamsters (*Phodopus sungorus*) are widely used as model organisms in studies of chronobiology, thermoregulation, and energy balance. They originate from the Siberian steppes, where environmental conditions vary widely across seasons (Ruf and Heldmaier 1992; Geiser et al. 2016). The upcoming winter is anticipated through decreasing day length, which serves as a reliable cue for seasonal acclimatization. When photoperiod falls below 13 h of light per day, the hamsters gradually adjust their behavior, morphology, and physiology to maintain energy balance under winter-like conditions (Figala et al. 1973; Steinlechner 2009).

**Fig. 1:**
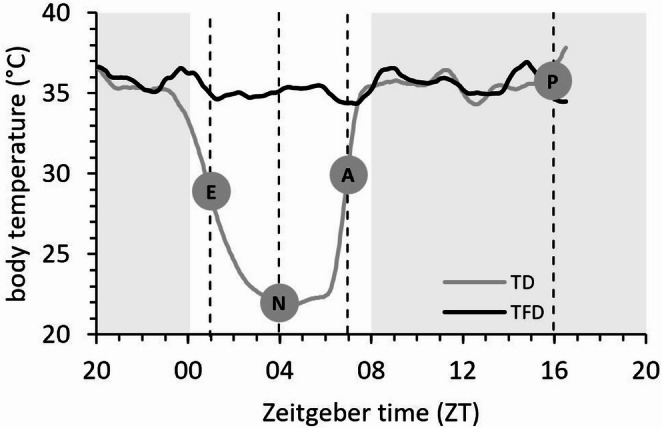
Sampling scheme over the course of one day. Time is indicated in hours after lights-on (Zeitgeber time, ZT00), the white area marks the light phase. Following the expression of at least one torpor bout, animals were sampled on a torpor day (TD, grey) during torpor entry (E; ZT01), torpor nadir (N; ZT04), torpor arousal (A; ZT07), or post-torpor (P; ZT16). On torpor-free days (TFD, black), animals were sampled at corresponding time points at normothermic body temperature. Background information on all individuals is provided in Supplementary Material 1.

During this photoperiodic acclimation, body temperature, locomotor activity, food intake, and body mass decline, reproductive organs regress and a dense winter fur develops (Figala et al. 1973; Steinlechner 2009). Once these coordinated adjustments to reduce energetic demands are complete, Djungarian hamsters exhibit spontaneous daily torpor as an additional energy-saving mechanism (Hoffmann 1973; Heldmaier and Steinlechner 1981a; Kauffman et al. 2001; Barrett et al. 2007; Haugg et al. 2021b). Notably, torpor occurs even at thermoneutrality, with *ad libitum* access to nesting material, food, and water. During torpor bouts, metabolic rate, and body temperature are markedly reduced for several hours during the resting (light) phase, while animals remain normothermic and active at night (Figala et al. 1973; Heldmaier and Steinlechner 1981b; Haugg et al. 2021b). The predictable timing of torpor enables comparison of distinct physiological phases during a torpor day (TD) with matched time points on a torpor-free day (TFD) (Ruf and Heldmaier 1992; Cubuk et al. 2017a; Haugg et al. 2021b) (Fig. 1).

Although torpor physiology has been studied in detail for a long time, the mechanisms controlling natural torpor remain poorly understood. During metabolic depression, transcription and translation are suppressed or reprogrammed while essential survival functions are maintained (Hittel and Storey 2002; Diaz et al. 2004; Heldmaier et al. 2004). Thus, torpor is not a passive response but an actively regulated process, likely coordinated within the brain. Accumulating evidence suggests that the hypothalamus plays a central role in torpor regulation (Cubuk et al. 2016; Jastroch et al. 2016; Diedrich et al. 2020). It integrates circadian, thermal, metabolic, and reproductive signals, key processes that are involved in photoperiodic acclimation and torpor expression (Ebling 2015; Buijs et al. 2016; Jastroch et al. 2016).

Transcriptomic analyses of the hypothalamus in Djungarian hamsters have revealed largely general responses during torpor entry and nadir, reflecting the downstream consequences of reduced metabolism and body temperature rather than the initiating regulatory signals (Cubuk et al. 2017a; Haugg et al. 2021a). This is likely due to the high anatomical and functional complexity of the hypothalamus, which comprises distinct nuclei and neuronal networks involved in diverse physiological processes. Consequently, sequencing of the whole hypothalamus may mask transcriptomic changes specific to individual functional units. Research in the thirteen-lined ground squirrel (*Ictidomys tridecemlineatus*) has demonstrated that transcriptional activity differs both spatially across hypothalamic nuclei and temporally across hibernation stages complemented by more recent studies identifying nucleus-specific circannual interval timing mechanisms in Djungarian hamsters (Bratincsak et al. 2007; Stewart et al. 2025). Together, these findings underscore the importance of examining discrete hypothalamic regions and neuronal populations, each of which plays specialized yet interactive roles in regulating metabolic and thermoregulatory processes.

In the present study, we aimed to identify hypothalamic nuclei that are differentially activated during defined states of a torpor bout compared with time-matched normothermic controls, using *c-Fos* as a marker of neuronal activity. Based on this activity mapping, we provide transcriptomic data from functionally relevant hypothalamic nuclei to further delineate neural structures and molecular pathways potentially involved in the regulation of natural daily torpor.

## Material & methods

### Animal work

#### Breeding and housing

Djungarian hamsters (*Phodopus sungorus*, Pallas 1773, Cricetidae, also known as Siberian hamster) were bred and raised at our own breeding colonies at the University of Hamburg and Ulm University, Germany (Reg.-Nr. 9/2014, z.231). The colonies were housed indoors in summer-like long photoperiod (16 h of light) provided by artificial light (150 lx). Constant red light (< 5 lx) enabled animal handling during the dark phase. From the age of six weeks, 71 hamsters were single-housed in Makrolon Type III cages (area: 820 cm^2^, height: 15 cm) equipped with wood shavings as bedding and tissue as nesting material at an ambient temperature of 19 ± 2°C. Tap water and food (hamster breeding diet 7014, Altromin Spezialfutter GmbH & Co. KG, Lage, Germany) were available *ad libitum*, supplemented by cucumber, oat flakes, and sunflower seeds once a week. One cohort of animals (*n* = 47) was used to assess transcriptional activity in distinct hypothalamic nuclei (experiment 1), the second cohort (*n* = 24) was used to generate transcriptome data of relevant hypothalamic nuclei (experiment 2).

#### Radiotelemetry

For both experiments 1 and 2, 71 adult (age 31.4 ± 4.8 weeks) male (*n* = 38) and female (*n* = 33) hamsters were transferred to winter-like short photoperiod (SP, 8 h of light). Background information for all animals is given in Supplementary File S1, Table 1 (experiment 1) and Table 2 (experiment 2). To continuously monitor core body temperature (Tb), all animals were implanted intraperitoneally with a radiofrequency transmitter (model TA-11TA-F10, silicone-coated, 1.1 cc volume, 1.6 g weight, 0.15 °C accuracy) under isoflurane anesthesia (2.5% and 1 ml/min for induction, 0.75 to 2.0% and 0.4 ml/min for maintenance, Forene^®^, AbbVie Deutschland GmbH & Co.KG, Ludwigshafen, Germany) and carprofen analgesia (5 mg/kg i.p. Rimadyl^®^, Zoetis Deutschland GmbH, Berlin). Recovery from surgery was supported by additional oat flakes, sunflower seeds, cucumber, and nesting material. Body mass, coat care, posture, and behavior were monitored daily for seven days until recovery. The PhysioTel™ radiotelemetry system with DataQuest™ ArtBronze software was used to record body temperature data from each hamster in 3-min-intervals (DSI - Data Sciences International, Harvard Bioscience Inc., St. Paul, MN, USA). A receiver board (RPC-1) was placed under each individual home cage to detect the transmitter radio signal that was collected by an exchange matrix (DEM) and transferred to a PC.

Experimental and surgical procedures were approved by the local animal welfare authorities (Authority for Health and Consumer Protection Hamburg, license 144_14; Regional Council Tübingen, license 1411).

#### Sampling scheme

Spontaneous daily torpor was defined as a body temperature < 32 °C for at least 30 min (Paul et al. 2005; Diedrich et al. 2015; Cubuk et al. 2017b). After each hamster had expressed at least one spontaneous daily torpor bout, animals were culled at different Zeitgeber times (ZT00 = lights on). For each sampling point (ZT01 = torpor entry (E), ZT04 = torpor nadir (N), ZT07 = torpor arousal (A), ZT16 = post-torpor (P), hamsters were sampled either on a torpor day (TD) or on a torpor-free day (TFD) (Fig. 1).

##### Experiment 1

To assess transcriptional activity in distinct hypothalamic nuclei, 47 hamsters (age 30.2 ± 4 weeks, 24 males, 23 females) were sampled after 16 ± 2 weeks acclimation to SP at ZT01-TD (*n* = 6, Tb 28.5 ± 2.2 °C), ZT01-TFD (*n* = 5, Tb 34.9 ± 0.4 °C), ZT04-TD (*n* = 6, Tb 23.5 ± 1.8 °C), ZT04-TDF (*n* = 5, Tb 35.4 ± 0.8 °C), ZT07-TD (*n* = 6, Tb 30.0 ± 1.8 °C), ZT07-TFD (*n* = 6, Tb 35.6 ± 1.0 °C), ZT16-TD (*n* = 7, Tb 36.0 ± 0.8 °C), ZT16-TDF (*n* = 6, Tb 35.9 ± 1.2 °C). Background information for all animals is given in Supplementary Material 1, Table 1.

##### Experiment 2

To generate transcriptome data from relevant hypothalamic nuclei during torpor entry and arousal, 24 hamsters (age 33.7 ± 5.4 weeks, 14 males, 10 females) were sampled after 14 ± 2 weeks acclimation to SP at ZT01 and ZT07 on a torpor day or a torpor free day ZT01-TD (*n* = 6, Tb 30 ± 0.3 °C), ZT01-TFD (*n* = 6, Tb 35 ± 1.3 °C), ZT07-TD (*n* = 6, Tb 30.3 ± 0.5 °C), ZT07-TFD (*n* = 6, Tb 35.9 ± 0.9 °C). Individual values are given in Supplementary Material 1 , Table 2.

In both experiments 1 and 2, the hamsters were culled with carbon dioxide in their home cage. After decapitation, the brain was dissected, frozen on dry ice, and stored at -80 °C. Radiotelemetry, body mass, and fur index data of 30 animals as well as small intestinal tissue of eleven animals have been analyzed previously (Haugg et al. 2021b; Piscitiello et al. 2021).

#### Cryosectioning

Coronal brain sections were cut from anterior to posterior, covering the hypothalamus from Bregma 0.02 mm to Bregma 3.04 mm (Franklin and Paxinos 2008). In experiment 1 (*n* = 47 animals), 16 μm thick sections were thaw-mounted on Poly-L-Lysine coated slides (Thermo Scientific, Walldorf, Germany). The sections were air-dried and stored at -80 °C for radioactive in situ hybridization of *c-Fos*. In experiment 2 (*n* = 24 animals), 20 μm thick sections were thaw-mounted on Poly-L-Lysine coated and UV-irradiated Carl Zeiss™ MembraneSlides 1.0 PEN (Fisher Scientific GmbH, Schwerte, Germany). The 72 sections per set were air-dried and used immediately for laser capture microdissection.

### Experiment 1: assessment of torpor-specific transcriptional activity in hypothalamic nuclei by *c-Fos* radioactive in situ hybridization

In situ hybridization was carried out as previously described (Bank et al. 2017). Brain sections were fixed in 4% PFA, washed in 0.1 M PBS, incubated with 0.25% acetic anhydrate dissolved in 0.1 M triethanolamine, washed in PBS, and dehydrated in an ascending ethanol series followed by vacuum drying. The riboprobe was synthesized, using T7 polymerase in the presence of 35 S-UTP from a cloned *c-Fos* DNA fragment (Morgan et al. 1996). The labelled probe was applied to slides in hybridization mixture (formamide, 0.3 M NaCl, 10 mM Tris–HCL (pH 8), 1 mM EDTA, 0.05% transfer RNA, 10 mM dithiothreitol, 0.02% Ficoll, 0.02% polyvinylpyrrolidone, 0.02% BSA, and 10% dextran sulphate) and hybridized at 58 °C overnight. Slides were washed in 4x saline-sodium citrate (SSC), incubated with ribonuclease A at 37 °C, washed in SSC solutions with decreasing concentrations, and dehydrated using graded ethanol. Dried slides were exposed to Kodak BioMax MR Films (Sigma-Aldrich Company Ltd., Poole, Dorset, UK) together with a 14 C microscale for 14 days.

Autoradiographs were scanned at 600 dpi and analyzed with Fiji ImageJ (1.52n Java 1.8.0_172 64-bit) (Schindelin et al. 2012). Integrated optical density (IOD) was calculated by reference to a standard curve [y = a + b × ln(x − c)]. Distinct *c-Fos* expression was observed in the suprachiasmatic nucleus (SCN), which was consequently defined and quantified as main area of interest. IOD was measured bilaterally in two sections per animal using a defined region of interest and averaged. Values per hamster are provided in Supplementary File S1 Table 1.

Statistics were performed using SigmaPlot version 15 (Systat Software, San Jose, CA, USA). Data were tested for normality and variance homogeneity in both groups (TD, TFD) per ZT using the Shapiro-Wilk and the Levene test, respectively. To assess the contribution of internal and external factors on *c-Fos* expression variability, a multiple linear regression was performed. In addition, the factor sex was evaluated as covariate for the two major factors group and ZT using an ANCOVA including an ANOVA interaction model and equal slopes assumption. Finally, *c-Fos* IOD was analyzed with a Two-Way ANOVA and Tukey *post hoc* test. Significance level was set to 0.05 with a power assumption of at least 0.8, correlation coefficients were rated as low |<0.3|, medium |0.3| to|0.5|, or high |>0.5|.

### Experiment 2: transcriptome analyses of SCN and PVN using laser capture microdissection and mRNA-Seq

Distinct *c-Fos* expression was observed in SCN during torpor. Therefore, we decided to laser capture and sequence neurons of the SCN and a well-known SCN output, the paraventricular nucleus (PVN). All solutions were prepared using RNAse-free water (incubation of 0.1% DEPC-Aqua dd. for at least 12 h at 37 °C, followed by autoclave) and absolute ethanol (200 Proof, Molecular Biology Grade, Fisher BioReagents™; Fisher Scientific GmbH, Schwerte, Germany). Using 50 ml plastic screw cap cells, each slide was dipped for one minute in 70% ethanol at -20 °C, for a half minute in 1% cresyl violet in 50% ethanol at 4 °C, for one minute in 70% ethanol at 4 °C, and for one minute in absolute ethanol at 4 °C, followed by air-drying. The cresyl-violet staining allowed to discriminate distinct nuclei using the mouse brain atlas as reference (Franklin and Paxinos 2008).

**Fig. 2:**
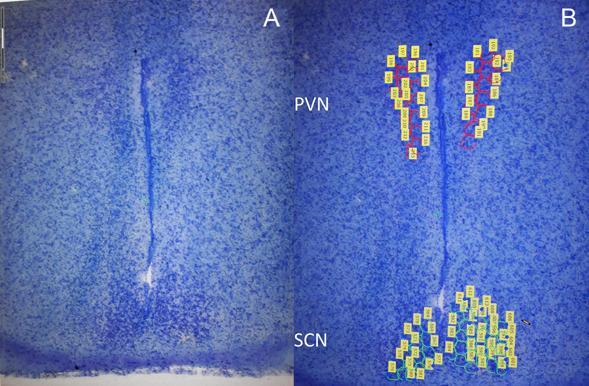
Laser capture microdissection of SCN and PVN. **A** Stained cell nuclei of one hypothalamic section including PVN and SCN. **B** Labelling of PVN and SCN prior to laser capture microdissection. The PVN (red) and the SCN (green) were labelled (yellow), cut along the circle-lines by the laser and blown into one tube per sample

Using the PALM MicroBeam with PALM RoboSoftware (Carl Zeiss Microscopy Deutschland GmbH, Oberkochen, Germany), SCN and PVN were separately laser-microdissected into a Carl Zeiss™ adhesive-cap tube (Fisher Scientific GmbH, Schwerte, Germany) filled with 50 µl RLT lysis buffer with 1:100 β-Mercaptoethanol, vortexed with lid downwards for 30 s, incubated with lid downwards on ice for 30 min, centrifuged at 12,000 rpm at room temperature for 5 min and stored at -80 °C (Fig. 2).

Total RNA of 48 samples (24 animals, 2 hypothalamic nuclei) was purified using the RNeasy Plus Micro Kit with gDNA eliminator spin column (Qiagen GmbH, Hilden, Germany) according to manufacturer’s instructions. For further processing, the samples were sent to StarSEQ GmbH, Mainz, Germany. Total RNA was checked for quality (Bioanalyzer, Agilent Technologies, Waldbronn, Germany) and quantity (RNA 6000 Pico Kit for very small RNA amounts of 50–5000pg/µl, Agilent Technologies, Waldbronn, Germany). RNA quality (RIN) of SCN and PVN was 8.3 ± 0.7 and 8.1 ± 0.7, the concentration ranged from 168 to 4349 pg/µl. The mRNA was isolated, the library prepared (NEBNext^®^ Single Cell / Low Input RNA library Prep Kit dual index), and the RNA-Seq performed on an Illumina NextSeq 500 (1 lane, 2 × 25 M reads, 2 × 150 nt, 7.5 gb). Individual values of quality and quantity are provided in Supplementary File S1 Tables 3 and 4.

### Data processing

The bioinformatic processing used in experiment 2 has previously been described (Haugg et al. 2023). Bioinformatics was performed on the bwForCluster NEMO of the Baden-Württemberg High Performance Computing (bwHPC) project. Raw Illumina read data was quality checked using FastQC 0.11.9^1^, adapter sequences and low-quality reads were trimmed using trim-galore 0.6.6^2^. The number of read pairs ranged from 13 to 58 M with an average of 36 M. Read pairs from all samples were pooled to generate a single *de novo* transcriptome assembly using Trinity 2.8.5 (Grabherr et al. 2013; Haas et al. 2013). In total, the *de novo* assembly included 1,748,627,781 read pairs, whereby SCN and PVN contributed equally with 890 M and 859 M read pairs, respectively (Supplementary File S1, Tables 3 and 4). The transcripts were mapped against the reference proteome of *Mus musculus* (GRCm39, Annotation Release 109)^3^ using blastx 2.5.0 + with an e-value < 1E-5 (Altschul et al. 1990; Genome Reference Consortium 2020a, b). A reduced assembly containing only transcripts with a hit against a mouse protein was generated. The reads from each sample were mapped back to the reduced assembly using bowtie 1.3.0 (Langmead 2010). Non-normalized differential gene expression per sample was calculated using rsem 1.3.1 (Li and Dewey 2011).

Raw and processed data of ZT01 were analyzed in the framework of a PhD thesis (Haugg 2022), and are accessible through GEO Series accession number GSE188129, including a *de novo* assembly of ZT01 not used in this study. Raw and processed data of ZT07 are accessible through GEO Series accession number GSE214011, including the *de novo* assembly of ZT01 and ZT07 used in this study. The data processing from raw Illumina data to non-normalized gene expression is provided in Supplementary Material 4.

GSE181298: https://www.ncbi.nlm.nih.gov/geo/query/acc.cgi?&acc=GSE181298

GSE214011: https://www.ncbi.nlm.nih.gov/geo/query/acc.cgi?acc=GSE214011

### Differential gene expression analyses

Differentially expressed genes (DEG) on a torpor day (TD) vs. a torpor free day (TFD) were detected using DESeq2 (Love et al. 2014). Statistical analyses were performed separately for SCN (Supplementary Material 5) and PVN (Supplementary Material 6). Only genes with a total read count of at ≥ 10 across all samples of a hypothalamic nucleus were analyzed. The statistical model design for each sampling point (ZT01 and ZT07) respected both, group (TD or TDF) and sex (male or female) of each hamster. The statistical pipeline comprised a normalization of data across all samples of each hypothalamic nucleus, a principal component analysis (PCA), and pairwise group comparisons (ZT01: TD vs. TFD; ZT07: TD vs. TFD).

The *de novo* assembly included a sample that was excluded from further analyses due to an outlying gene expression profile of the SCN sample.

Mouse GeneIDs were assigned with blastx and used to aggregate calculated transcript expression values at gene level. Each GeneID includes all isoforms, precursors, and preproproteins of that gene. The pairwise comparison of the normalized counts per group resulted in the gene’s fold change, provided as log2(FC), and the significance of this fold change (adjusted p-value, padj). Genes with a padj < 0.05 were defined as differentially expressed genes. Fold changes are given for the first named group relative to the second named group (Supplementary Material 2).

To identify overrepresented reactome pathways (Reactome version 77, released 2021-10-01), GO overrepresentation analyses^4^ were performed with Fisher’s Exact as test type and False Discovery Rate (FDR) correction (The Gene Ontology Consortium et al. 2000, 2021; Mi et al. 2019). For each pairwise group comparison, increased (padj < 0.05 and log2(FC) > 0) and decreased DEGs (padj < 0.05 and log2(FC) < 0) were tested separately to increase the informative value, since the test itself does not consider the direction of regulation (Hong et al. 2013). All present genes (any padj-value, any log2(FC)-value) served as background. Hierarchical ranking of DEG-driven pathways was based on the information on the Reactome Homepage^5^ (Gillespie et al. 2022).

## Results

### Experiment 1: transcriptional activity was increased during torpor in the suprachiasmatic nucleus (SCN)

Autoradiographs revealed a clear *c-Fos* signal in the SCN, but not in other hypothalamic nuclei.

**Fig. 3:**
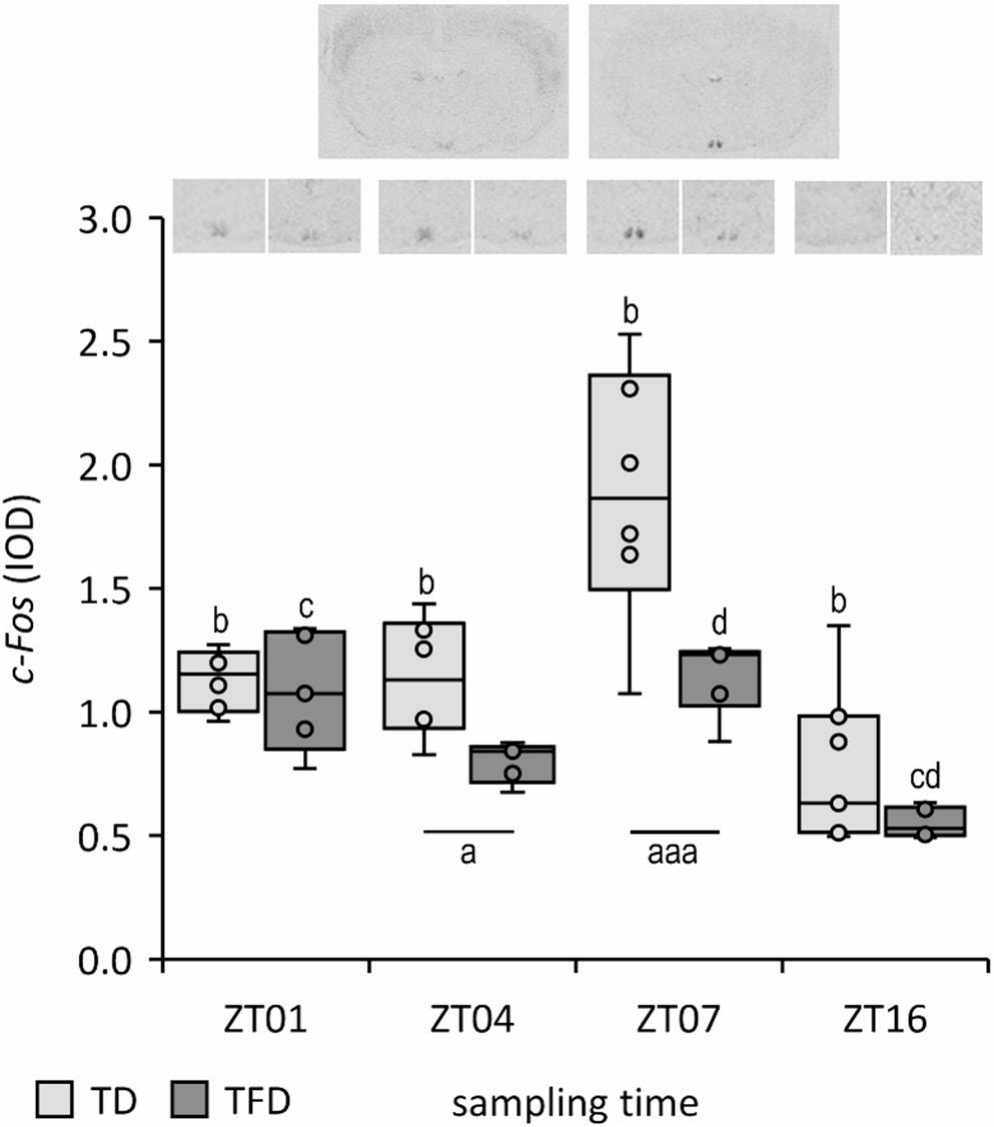
Radioactive in situ hybridization of *c-Fos* and quantification in the SCN. The IOD differed between torpor days (TD) and torpor-free days (TFD) (Two-Way ANOVA for ZT F_(3,39)_ = 22.5, *p* < 0.001, power = 1.0; group F_(1,39)_ = 18.5, *p* < 0.001, power = 1.0; Interaction F_(3,39)_ = 3.5 *p* = 0.024, power = 0.6, Tukey *post hoc* test) at ZT04 (^a^*p*=0.04) and ZT07 (^aaa^*p*<0.001). Further ZT-related significant differences (*p* < 0.01) are indicated with the same letters (b, c,d). Values per animal are provided in Supplementary Material 1.

The *c-Fos* IOD in the SCN was higher during torpor nadir at ZT04 and torpor arousal at ZT07, than on a TFD (Fig. 3). Consequently, we decided to analyze the transcriptome of SCN and it´s well known output PVN during torpor entry and arousal.

Within torpor days, *c-Fos* IOD was significantly higher at ZT07 compared to all other ZTs. On torpor free days, *c-Fos* IOD was significantly lower at ZT16 compared to ZT01 and ZT07. Sex did not affect the influence of ZT on *c-Fos* expression (ANOVA Interaction Model F_(3,39)_ = 2.1, *p* = 0.110, ANOVA Equal Slopes F_(1,42)_ = 0.4, *p* = 0.549) but values grouped as TD or TFD were influenced by sex as covariate (ANOVA Interaction Model F_(1,43)_ = 5.1, *p* = 0.029). Two-way ANOVA confirmed that sex alone did not contribute to the observed variation, and the interaction power between group and sex indicated that any effect of sex could be detected with only a 50% probability (group F_(1,43)_ = 6.7, *p* = 0.013, power = 0.7; sex F_(1,43)_ = 0.3, *p* = 0.603, power = 0.0; Interaction F_(1,43)_ = 5.1 *p* = 0.029, power = 0.5). Thus, the effect of sex was considered to be of minor importance.

### Experiment 2: Transcriptomics of SCN and PVN during torpor entry and arousal

#### *De novo* assembly and mapping

An average of 36 M read pairs were sequenced per nucleus. Overall, the *de novo* assembly of transcriptomes included 1,748,627,781 read pairs from all samples of SCN and PVN. Using a blastx search against the mouse proteome, 15,888 and 15,976 distinct protein coding genes were identified in the transcriptome data of the SCN and the PVN, respectively, corresponding to approximately 72% of the entire gene repertoire of the mouse (22,177 protein-coding genes) for both nuclei.

#### Differential gene expression and enriched reactome pathways

**Fig. 4:**
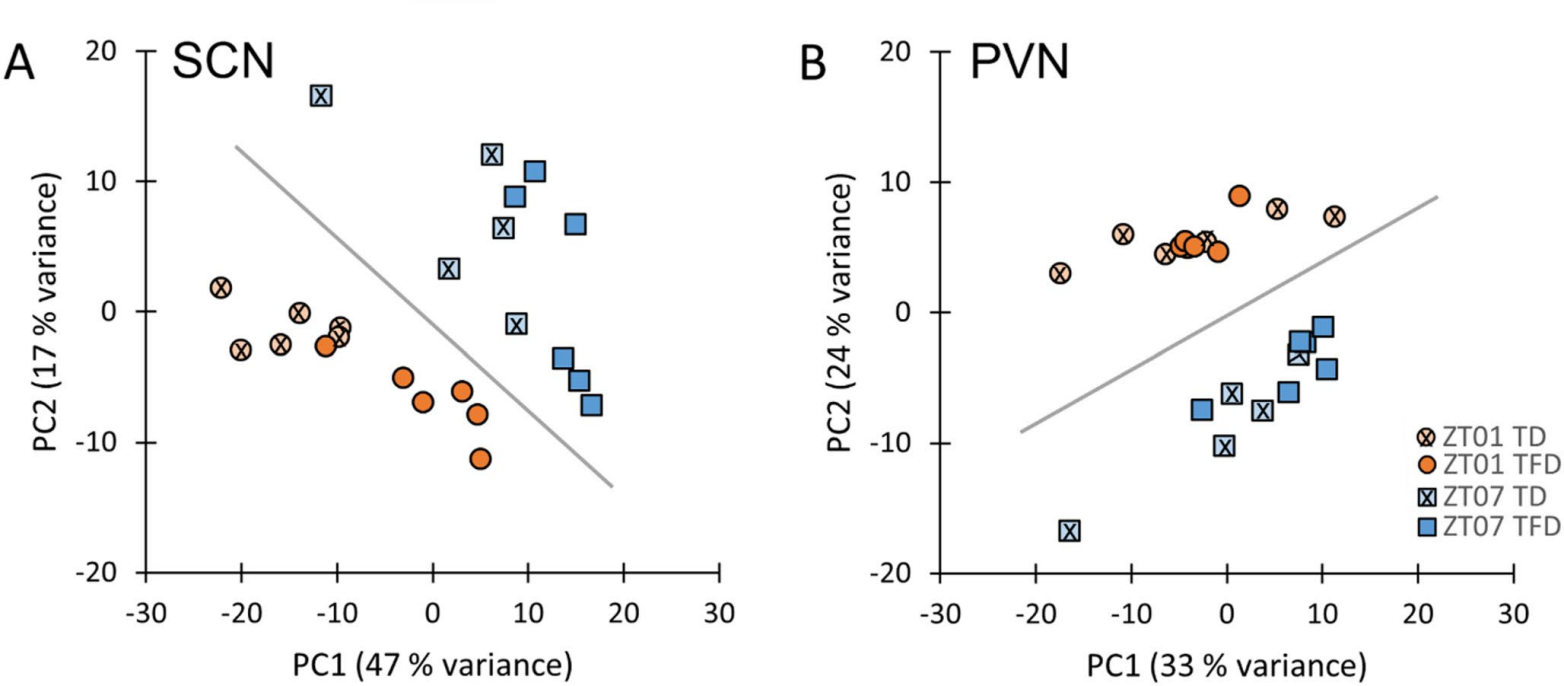
Principal component analysis of SCN (A) as well as PVN (B) sampled at ZT01 (orange circle) or ZT07 (blue square) on a torpor-day (TD, light) or a torpor-free day (TFD, dark).

The principal component analyses showed distinct clustering by time point (ZT01 vs. ZT07) in both SCN and PVN, emphasizing the importance of circadian time as sampling factor (Fig. 4A, B). Within ZT01, samples of TD were separated from samples of TFD in the first and second dimension in SCN, except for one sample from TFD that intermixed with the cluster of TD. Within ZT07, TD and TFD were separated in the first dimension, but overlapped in the second dimension in SCN (Fig. 4A). In the PVN, TFD samples clustered closely in both dimensions at ZT01, whereas TD samples spanned a wider range in the first dimension. At ZT07, TD and TFD samples were largely intermixed (Fig. 4B).

**Fig. 5:**
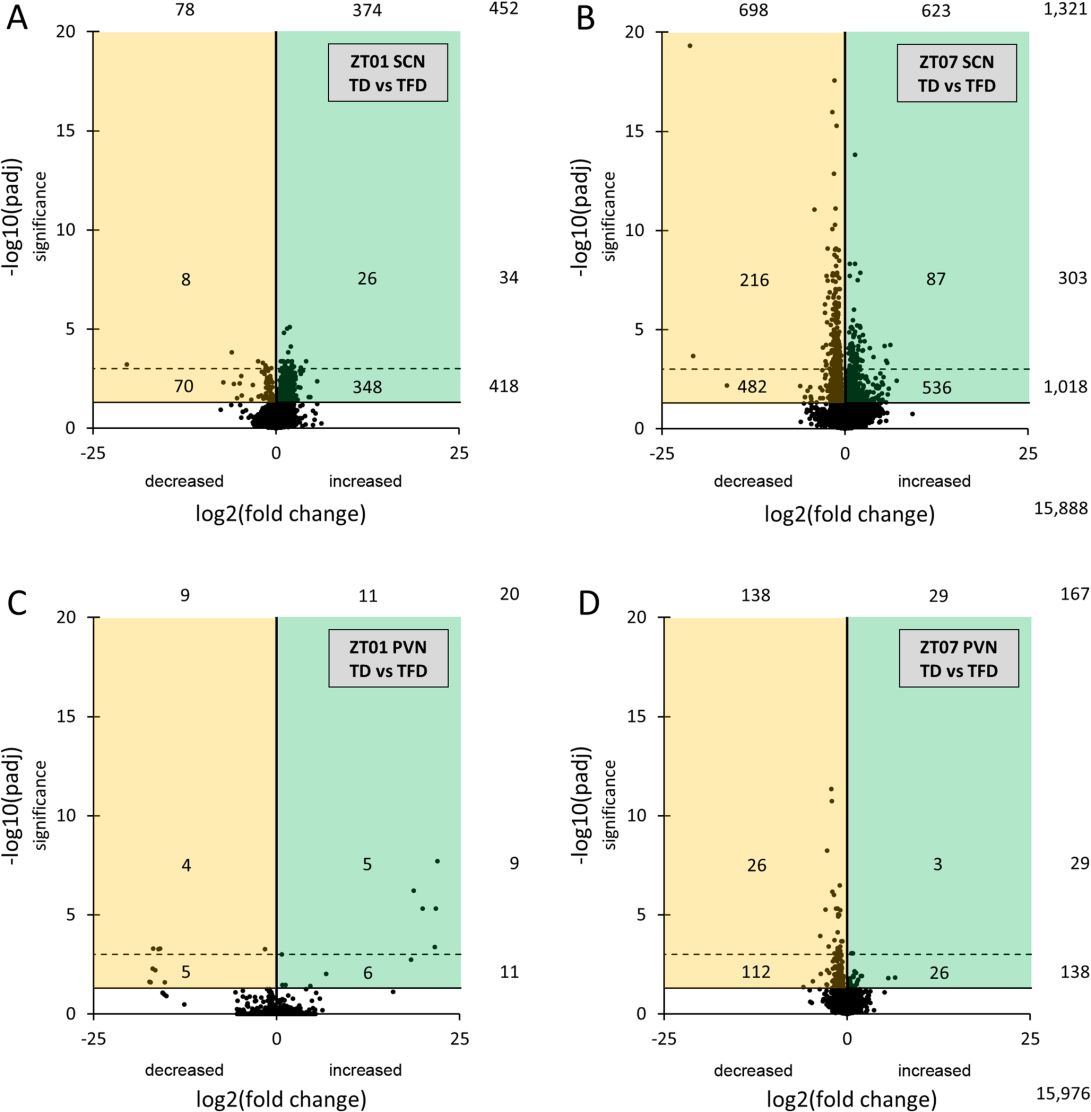
Volcano plots. Differential gene expression for the pairwise group comparisons torpor-day (TD) vs. torpor-free day (TFD) of SCN (A + B) and PVN (C + D) sampled at ZT01 (A + C) or ZT07 (B + D). DEGs (padj < 0.05) are shown above the horizontal solid line (-log10(0.05) = 1.3), DEGs with padj < 0.001 are shown above the dotted line (-log10(0.001) = 3.0). Genes with decreased expression are shown within the yellow area, genes with increased expression within the green area. The number DEGs are indicated per area, the number in the right upper corner states the total number of regulated genes, the number in the right lower corner the total number of annotated genes. All genes are listed in Supplementary Material 2.

Overall, more genes were differentially expressed in SCN than PVN at both sampling times. In both nuclei, less genes were differentially expressed during torpor entry at ZT01 than during torpor arousal at ZT07 (Fig. 5).

In SCN, 452 genes were differentially expressed at ZT01. This accounts for 2.8% of identified genes. 374 of these DEGs showed and increased and 78 a decreased expression, accounting for an up-to-down-ratio of 4:1. The DEGs included genes involved in transcription (*FosB*,* JunB)* and the circadian clockwork (*Avp*,* Timeless*). Three reactome pathways associated with “Metabolism of proteins”, i.e., “Mitochondrial translation”, “Mitochondrial translation elongation”, and “Mitochondrial translation termination”, were overrepresented by the increased DEGs, no pathway was overrepresented by decreased DEGs (Tables 1 and 2, Supplementary Material 3 ).

**Table 1:** Potentially interesting DEGs in SCN

		System	GeneID	Gene	Gene product	Padj	-log10(padj)	log2(FC)
SCN	ZT01	Transcription	14,282	*Fosb*	protein fosB	0.0024	2.6	-4.74
			16,477	*Junb*	transcription factor jun-B	0.0384	1.4	2.99
		Clockwork	11,998	*Avp*	vasopressin-neurophysin 2-copeptin	0.0061	2.2	2.55
			21,853	*Timeless*	timeless circadian clock 1	0.0046	2.3	1.27
	ZT07	Transcription	14,281	*Fos*	proto-oncogene c-Fos	0.0124	1.9	1.12
			16,477	*Junb*	transcription factor jun-B	0.0061	2.2	3.81
		Clockwork	11,865	*Bmal1*	brain and muscle ARNT-like 1	0.0001	4.0	-0.89
			20,893	*Bhlhe40*	class E basic helix-loop-helix protein 40	0.0022	2.7	0.61
			17,773	*Mtnr1a*	melatonin receptor type 1 A	0.0004	3.4	-2.67
			21,853	*Timeless*	timeless circadian clock 1	0.0362	1.4	1.02
			20,604	*Sst*	somatostatin	0.0021	2.7	2.38

Gene expression of genes involved in transcription and circadian clockwork were increased (green) or decreased (yellow) around ZT01 (orange) and ZT07 (blue) in the SCN. Results of all mapped genes are provided in Supplementary File S2

**Table 2:** Enriched reactome pathways

Brain area	Sampling time	Gene subset	FDR	Reactome pathway	
SCN	ZT01	down	–	–	–
		Up	n.s.	R-MMU-392,499	Metabolism of proteins
			9.41E-03	R-MMU-5,368,287	Mitochondrial translation
			2.09E-02	R-MMU-5,389,840	Mitochondrial translation elongation
			1.28E-02	R-MMU-5,419,276	Mitochondrial translation termination
	ZT07	Down	6.89E-10	R-MMU-74,160	Gene expression (Transcription)
			1.97E-04	R-MMU-212,436	Generic Transcription Pathway
			4.79E-07	R-MMU-73,857	RNA Polymerase II Transcription
			1.61E-03	R-MMU-6,807,505	RNA polymerase II transcribes snRNA genes
		Up	–	–	–
PVN	ZT01	Down	–	–	–
		Up	–	–	–
	ZT07	Down	3.35E-05	R-MMU-74,160	Gene expression (Transcription)
			3.82E-02	R-MMU-212,436	Generic Transcription Pathway
			1.53E-03	R-MMU-73,857	RNA Polymerase II Transcription
			4.21E-02	R-MMU-426,496	Post-transcriptional silencing by small RNAs
			3.06E-02	R-MMU-4,839,726	Chromatin organization
			3.67E-02	R-MMU-3,247,509	Chromatin modifying enzymes
		Up	–	–	–

Pathways enriched by the subsets of decreased or increased DEGs for each of the four pairwise group comparisons of TD vs. TFD (SCN-ZT01, SCN-ZT07, PVN-ZT01, PVN-ZT07). All 15,888 and 15,976 genes identified in SCN and PVN, respectively, served as respective background. All enriched pathways were overrepresented. Three pathways were driven by decreased DEGs in both SCN and PVN at ZT07 (red). Pathways were sorted in blocks according to each hierarchically highest pathway (bold) with its sub-pathways (each hierarchical level indicated with “-“). Pathways with false discovery rate (FDR) < 0.05 were taken as significant. The pathway indicated as not significant (n.s., grey) had an FDR > 0.05 and is serving for orientation. Comprehensive results are given in Supplementary File S3

In SCN at ZT07, 1,321 genes (8.3%) were differentially expressed (623 increased and 698 decreased, ratio 1:1), including transcription factors (*c-Fos*,* Junb)*, clock genes (*Bmal1*,* Mtnr1a*,* Bhlhe40*,* Timeless*,* Sst)*. The reactome pathway “Gene expression (transcription)” and three sub-pathways, i.e., “Generic Transcription pathway”, “RNA Polymerase II Transcription”, and “RNA Polymerase II transcribes snRNA genes”, were overrepresented by the downregulated DEGs (1 and 2, Supplementary File S3).

In PVN at ZT01, 20 genes (0.1%= were differentially expressed (11 increased and 9 decreased, ratio 1.5:1). Functions of the DEGs were diverse and partly unknown and subsets of up- and downregulated genes did not show enrichment for reactome pathways (Supplementary Material 3, Table 2).

In PVN at ZT07, 167 genes (2.8%) were differentially expressed (29 increased and 138 decreased, ratio 1:4). The decreased DEGs were enriched in the reactome pathways “Gene expression (Transcription)” and three sub-pathways, i.e., “Generic Transcription Pathway”, “RNA Polymerase II Transcription”, and “Post-transcriptional silencing by small RNAs”, as well as “Chromatin organization” and one sub-pathway “Chromatin modifying enzymes” (*Supplementary* Material 3 , Table 2).

## Discussion

In our study, radioactive in situ hybridization of *c-Fos* revealed distinct and differential transcriptional activation of SCN during torpor, relative to samples of time-matched normothermic animals, that was increasing over torpor duration. This increase is consistent with studies of deep hibernators, showing low SCN *c-Fos* levels during torpor entry that are increasing over the course of the bout and peaking during arousal, emphasizing a critical role in timing of torpor bouts and reinstatement of circadian rhythmicity during normothermic periods (Bratincsak et al. 2007; Revel et al. 2007). In the Djungarian hamster, spontaneous daily torpor is precisely timed into the resting (light) phase of the circadian cycle and behavioral as well as lesion and molecular studies have demonstrated that the clock is critical for the timely organization of torpor expression (Ruby and Zucker 1992; Ruf and Heldmaier 1992; Herwig et al. 2007; Cubuk et al. 2017b). Given the prominent and distinctive *c-Fos* expression and the robust circadian organization of daily torpor in this species, our primary focus was to generate torpor specific transcriptomes of SCN during torpor entry and arousal to narrow down potential signalling mechanisms involved. Ablation of the SCN leads to a loss of temporal torpor organization, but the overall capacity for torpor remains, indicating that SCN is critical for gating but not for torpor generation (Ruby and Zucker 1992). A prominent output region of the SCN is the PVN. Direct neural projections allow the SCN to convey circadian timing signals to the PVN, which then orchestrates a range of crucial physiological processes (Leak and Moore 2001; Santoso et al. 2018). There is clear evidence for a role of the PVN in torpor control in different rodent species, including activation at torpor onset in mice as well as significant loss of spontaneous torpor expression after ablation in Djungarian hamsters (Ruby 1995; Hitrec et al. 2019; Hare et al. 2024), Consequently, we decided to add the PVN transcriptome to our study, hypothesizing that SCN might generate an output signal to PVN that in turn regulates torpor entry and/or arousal.

### Transcriptomics of SCN and PVN during torpor entry and arousal

#### Differential gene expression and pathways

Comparable numbers of genes were annotated in SCN and PVN, but more genes were differentially expressed in SCN than in PVN during both, entry (452 and 20) and arousal (1,321 and 167), suggesting a more dynamic role for SCN in daily torpor control. In both SCN and PVN, more genes were differentially expressed during arousal than during entry, likely reflecting the rigorous and quick transition from torpor to active state that is completed within approximately 40 min in this species (Mertens et al. 2008).

The principal component analyses showed distinct clustering of time points (ZT01 vs. ZT07) in both SCN and PVN (Fig. 4A, B), demonstrating distinct transcriptome profiles at the different times of day. This finding is logical for the circadian clock and closely related structures such as PVN and emphasizes the importance of circadian matched normothermic control animals whenever possible. In the SCN, samples were largely separated according to group (TD vs. TFD) at ZT01 and ZT07, strongly indicating a torpor specific gene activation during entry as well as arousal in this nucleus (Fig. 4A). During torpor entry, increased DEGs in the SCN were significantly enriched in pathways associated with protein metabolism, particularly mitochondrial translation, suggesting enhanced mitochondrial metabolism and hence energy production during this phase. Although mitochondrial metabolism has been shown to be decreased during torpor, mitochondrial metabolic adaptations have been shown to be highly dependent on tissue type, species and the nature of torpor (Staples and Brown 2008). In Djungarian hamsters, mitochondrial respiration has been shown to be depressed during daily torpor in the liver, but not in kidney, skeletal muscle and heart (Kutschke et al. 2013). However, selective enhancements in mitochondrial functions in the liver including increased fatty acid oxidation and TCA cycle enzymes, suggest increased mitochondrial capacity for substrate oxidation, energy transfer, and uncoupling processes that could support controlled thermogenesis and ROS management (Kovacs et al. 2025).

Enhanced mitochondrial translation in the SCN during torpor entry supports the compelling evidence that the SCN remains functionally active during torpor, even when most other brain regions exhibit reductions of activity. In Djungarian hamsters, rhythmic expression of clock genes during daily and fasting-induced torpor has been demonstrated (Herwig et al. 2007; Cubuk et al. 2017b), illustrating that the clockwork remains largely functional during the short torpor bouts at moderate body temperatures above 15 °C. Although clock genes are not rhythmically expressed in deep hibernators at body temperatures well below 15 °C (Revel et al. 2007; Ikeno et al. 2017), multiple studies have shown hibernation phase-dependent increased SCN activity, peaking prior to arousal as well as remarkable temperature compensation (Kilduff et al. 1989, 1990; Bitting et al. 1994; Ruby and Heller 1996; Bratincsak et al. 2007; Revel et al. 2007). Collectively, these findings support the view that the SCN possesses specialized molecular and cellular mechanisms that enable it to function and coordinate timely torpor organization, even when overall brain activity is greatly suppressed.

At ZT01, we found upregulation of *Timeless* and *Avp in* SCN during torpor entry. Although the precise role of timeless in the mammalian circadian system remains debated, it has been proposed to support circadian regulation by fine tuning clock protein stability and phase responsiveness (Kurien et al. 2019). Moreover, timeless has been shown to contribute to the DNA damage response to replication stress, hence may aid to maintain functional integrity of SCN during torpor (Cai and Chiu 2022). *Avp* is a well-known clock output signal that has been shown to relay light reception to inhibit feeding behaviour by activating oxytocin neurons in the PVN in rats (Santoso et al. 2018). This is particularly interesting, since a recent study has demonstrated that activation of oxytocinergic neurons in the PVN enhances torpor duration and depth in mice (Hare et al. 2024). Hence, it is very tempting to speculate, that *Avp* output from SCN might enhance activation of oxytocin neurons in the PVN and thereby induce or facilitate spontaneous torpor in Djungarian hamsters. The only DEG known to be involved in modulating activity of PVN oxytocin neurons in our dataset is *Cyp19a1*, that was decreased in PVN at ZT01. *Cyp19a1* encodes aromatase that can locally convert androgens to estrogens, influencing oxytocin release (Spool et al. 2022). Although a decrease of *Cyp19a1* is counterintuitive in this context, it might be explained by increased translation and therefore less mRNA and requires investigation at protein level.

Overall, we found only 20 DEGs during torpor entry in PVN that did not clearly separate in the PCA and did not enrich pathways. Their partly unknown functions or multifunctionality complicates direct functional interpretation in this region-specific transcriptomic dataset. This does not exclude distinct functional changes driven by few genes, but requires more detailed analyses at protein and network level in the future. Moreover, seasonal shifts in PVN specific gene expression may gate or modulate torpor propensity (Stewart et al. 2025).

During torpor arousal, the respective subset of downregulated genes caused an overrepresentation of “gene expression (transcription)” and sub-pathways in both SCN and PVN. The subset of genes downregulated in PVN drive an overrepresentation of “chromatin organization” and its sub-pathway “chromatin modifying enzymes”. Decreased transcription during arousal is somewhat surprising, since most studies suggest that during arousal from torpor gene transcription is reactivated and suppression largely occurring during torpor itself.

During both deep and daily torpor, global gene transcription rates drop across multiple tissues including the hypothalamus, liver, and brown adipose tissue (van Breukelen and Martin 2002; Diaz et al. 2004). This depression has been associated with epigenetic changes — such as reduced histone acetylation and elevated transcriptional repressors — that are reversed upon arousal (Biggar and Storey 2014; Tessier et al. 2017). Nevertheless, some genes, including several protein phosphatase 1 regulatory subunits involved in glycogen synthesis and carbohydrate metabolism, show reduced nascent transcription during torpor and remain suppressed during arousal relative to the summer-active state in Syrian hamster livers (Coussement et al. 2023), consistent with the metabolic shift that occurs during rewarming.

Although transcription slows, transcripts initiated at torpor onset can accumulate throughout the hypometabolic phase. As animals enter arousal, transcription and translation rebound, supporting cellular recovery (Gillen et al. 2021). Recent studies further show that transcripts stabilized during torpor are typically degraded or diluted during arousal, marking the shutdown of torpor-specific transcriptional programs as normal metabolism resumes (Grabek et al. 2015; Fu et al. 2021). Moreover, distinct activation of transcription factors such as *cFos* and *Junb* expression in the hypothalamus during arousal, as well as distinct control of several genes involved in the clockwork is in line with other data and emphasizes distinct torpor phase and site-specific transcriptional control. In SCN, this is likely contributing to maintain accurate timekeeping (Bratincsak et al. 2007; Revel et al. 2007; Herwig et al. 2007; Fu et al. 2021; Haugg et al. 2023).

It has to be taken into account that in our study, the animals were sampled during arousal and compared to a normothermic group at the same Zeitgeber time, a setup that is not possible in deep hibernators in which long torpor bouts are interrupted by brief normothermic intervals and most studies have focused on comparing deep torpor states to fully aroused state. Even if samples are taken during the actual arousal, the comparison to the normothermic group is different from our setup since circadian phase cannot be taken into account since no animals remain normothermic over the time of other animals’ entire deep torpor bouts like in daily heterotherms.

Hence, although decreased transcription during arousal appears counterintuitive given the general reactivation of gene expression at this stage, our findings highlight strong phase- and region-specific regulation. Together with evidence that global transcriptional depression occurs mainly during torpor, that some metabolic genes remain suppressed into arousal, and that torpor-stabilized transcripts are rapidly cleared as metabolism resumes, these results underscore the complexity of transcriptional transitions between torpor and arousal.

## Conclusion

Our findings show that the SCN exhibits robust, torpor-specific transcriptional regulation underscoring its central role in timing daily torpor in Djungarian hamsters. In contrast, the PVN displays far fewer transcriptional changes, suggesting more limited but potentially targeted involvement. Enhanced mitochondrial translation and clock-related gene activation in the SCN support its functional resilience during hypometabolism, while phase-specific suppression of broader transcriptional programs in both nuclei highlights distinct regulatory dynamics between entry and arousal. Together, these data identify the SCN as a primary driver of torpor organization and point to SCN–PVN signalling as a promising pathway for future mechanistic investigation.

## Supplementary Information

Below is the link to the electronic supplementary material.

Supplementary Material 1

Supplementary Material 2

Supplementary Material 3

Supplementary Material 4

Supplementary Material 5

Supplementary Material 6

## Abbreviations

DEG: Differentially expressed gene
GO: Gene ontology
IOD: Integrated optic density
log2(FC): Logarithm of a gene's fold change to the base 2
padj: Adjusted p-value of a gene's expression
PCA: Principal component analysis
PVN: Paraventricular nucleus
SCN: Suprachiasmatic nucleus
SP: Short photoperiod
Tb: Body temperature
TFD: Torpor-free day, defined by normothermia with Tb > 32 °C
TD: Torpor-day, defined by hypothermia with Tb < 32 °C for at least 30 min
ZT: Zeitgeber-time, here: hours after lights on at ZT00

## Footnotes

1
https://www.bioinformatics.babraham.ac.uk/projects/fastqc/.

2
https://github.com/FelixKrueger/TrimGalore.

3
https://www.ncbi.nlm.nih.gov/books/NBK279688/.

4
http://pantherdb.org/tools/compareToRefList.jsp.

5
https://reactome.org/.
